# Institutional influence on length of stay in German forensic hospitals: a multilevel analysis of patients with schizophrenia spectrum disorders

**DOI:** 10.3389/fpsyt.2024.1456363

**Published:** 2024-10-17

**Authors:** Lukas Stürner, Thomas Ross, Jan Querengässer, Hans-Joachim Traub

**Affiliations:** ^1^ Department for Psychiatry and Psychotherapy I, Faculty of Medicine, Ulm University, Ulm, Germany; ^2^ Clinic for Forensic Psychiatry, Weissenau Psychiatric Centre, Ravensburg, Germany; ^3^ Clinic for Forensic Psychiatry and Psychotherapy, Reichenau Psychiatric Centre, Reichenau, Germany; ^4^ Clinic for Psychosomatic Medicine and Psychotherapy, Faculty of Medicine, University of Ulm, Ulm, Germany; ^5^ LVR-Institute for Research and Education - Section Healthcare Research, Cologne, Germany; ^6^ Department of Psychology, FernUniversität in Hagen, Hagen, Germany

**Keywords:** mental disorder, forensic psychiatry, linear mixed models, hospital effect, forensic psychotherapy, multi-center study

## Abstract

**Introduction:**

Scientific studies have focused on patient-related characteristics as predictors of length of stay in forensic psychiatry. However, little attention has been paid to the specificities of forensic psychiatric settings. This study aims to test whether differences in forensic admissions transcend individual factors by comparing length of stay between different psychiatric units, controlling for hospital characteristics and patient characteristics.

**Methods:**

The dataset was derived from a forensic documentation system containing a wide range of information on forensic psychiatric patients. N = 594 patients with schizophrenia spectrum disorders discharged from 6 forensic hospitals in southern Germany were included in a linear mixed regression model. Linear mixed models were calculated, allowing for the simultaneous estimation of variance between patients and hospitals.

**Results:**

The final regression model explained 49% of the total variance. The only statistically significant patient-related predictors were age at admission, education and severity of the index offence. Hospital differences explained 41% (ICC) of the variance in length of stay without finding a significant hospital effect in the data.

**Discussion:**

Previous research has primarily analyzed predictors of length of stay in terms of individual patient characteristics. This work suggests that variables other than patient-related factors need to be considered when assessing the length of stay in forensic units. Further multi-center studies are needed to gain a better understanding of how forensic psychiatric hospitals and other institutional influences affect length of stay.

## 1 Introduction

Forensic mental health care has to meet two often conflicting demands: the treatment of mentally disordered offenders and their reintegration into society, and the protection of the community from the potential threat of future crime. Responsibility for forensic mental health care varies across the EU. Within the basic legal framework, forensic issues are mainly regulated by health or criminal law (1–4). This indicates whether the issue is seen primarily as a matter of clinical care or a matter of public safety. Psychiatric treatment is offered to criminally responsible offenders in prison, who receive psychiatric consultations on a voluntary basis. Forensic treatment is provided to offenders who, due to their mental disorder, are considered to have diminished or no responsibility for their crime and who are at high risk of reoffending. The manner and extent to which forensic psychiatric patients are treated ultimately depends on the jurisprudence of a country. Therefore, it is challenging to make direct comparisons between different forensic systems in different countries. For example, the average length of stay varies considerably across EU countries, from 1.04 years in Slovenia to 10 years in the Netherlands (4). An understanding of a country’s forensic system is therefore essential for the evaluation of empirical evidence.

### 1.1 Forensic psychiatry in Germany

In Germany, admission to a forensic psychiatric unit (Section 63 of the German Criminal Code) requires three conditions to be met: [1] the commission of a criminal offence, [2] the presence of a mental illness, disorder or intellectual disability that is certain to substantially diminish the offender’s responsibility, and [3] the predominant likelihood that the offender will commit further serious offences as a result of his or her condition. Federal state regulations outline both patient rights and staff responsibilities and vary from state to state, with almost no common procedures except for the admission and discharge of forensic patients. There are about 65 forensic psychiatric hospitals in all states, ranging from high security to open wards. Discharge is recommended when the patient is no longer likely to commit a serious crime. Legislation requires that the appropriateness of a forensic placement be reviewed iteratively, while the length of placement can be theoretically and practically unlimited. The decision to release a patient is taken by the court in cooperation with a prosecutor, the patient’s lawyer, the forensic psychiatric unit and, at least periodically, an external expert. This decision is based solely on the patient’s ability to no longer pose a threat to society. In certain cases, the duration of inpatient forensic treatment may exceed the maximum sentence that would be imposed for similar offences committed by individuals without a diagnosed mental disorder. Although there is no empirical evidence to support this assumption, it is a reasonable assertion based on the inherently open-ended nature of forensic treatment. Germany has the second highest forensic prevalence rate in the EU, with 12.6 forensic psychiatric beds per 100,000 inhabitants (3). Furthermore, the number of patients treated in forensic psychiatric hospitals has been on the rise for over three decades (5). The greatest challenge currently facing forensic psychiatric care in Germany is the treatment of patients with schizophrenia spectrum disorders (SSD), largely due to the significant increase in their numbers. In the state of Baden-Württemberg (BW), this group of patients now constitutes between 70 and 80 percent of the total forensic psychiatric population (5, 6).

### 1.2 Factors influencing the length of stay

Studies examining length of stay in forensic psychiatry have employed different approaches. The two main quantitative methods used in the literature are multiple linear regression and logistic regression analyses. However, it is important to note that the definition of length of stay varies across studies (7). Many studies focus on identifying individual factors that contribute to patients being classified as long-stay. A less common approach involves interviewing experts or inpatients.

The results of quantitative studies reveal a large number of predictors, although these are not always consistent. A study in Ireland found that men tend to stay longer in forensic hospitals than women (8). A study in the Czech Republic identified other factors that may influence length of stay, including older age, being in a relationship, and being employed prior to admission (9). In the United Kingdom, another study found that long-stay patients tend to have a higher level of education than short-stay patients (10). However, other studies have found no predictive influence of socio-demographic characteristics at all (11–14). The authors’ findings strongly suggest that long and frequent previous contact with psychiatric services (13, 15), the presence of persistent psychotic and delusional symptoms (16, 17), early age at first documented offence (18), escape attempts or absconding (15, 16) and high scores on the HCR-20 (16, 17) are important factors associated with length of stay in forensic psychiatric treatment. Another area of contention is the relationship between substance use disorders and length of stay. Some studies have indicated that individuals with substance use disorders may require longer stays in forensic hospitals (15). However, other studies have found the opposite effect (16) or no significant evidence (8, 19). In a 2016 systematic review (20), results from nine out of ten studies showed a positive association between index offence severity and length of stay. A Swiss study (14) found that homicide and sexual abuse had the greatest impact on length of stay. Some studies have confirmed the influence of psychiatric diagnosis on length of stay (12–15), while others have rejected this influence (8, 19, 21). One reason for the heterogeneity of results is that each country follows a different path and defines access to forensic psychiatry differently.

With regard to the influence of institutions or differences between hospitals, the only study in the literature that addresses this issue is a comparison of seven different regions of medium secure forensic psychiatric services in the UK (22). The average length of stay varied between the regions. The authors attribute these differences between regions to their resources and prioritization of services, given that patients with mental illness differ between regions. All conclusions are based on descriptive statistics only. It is worth mentioning a German study that controls for institutional influence on the increase in forensic hospital admissions (23). The difference in inpatient growth can be attributed to higher rates of sentencing by regional courts, with no significant differences in clinical-forensic patient characteristics or discharge rates.

In a qualitative study by Connell et al. (24), forensic experts from 16 European countries hypothesized that deinstitutionalization in general mental health in the 1970s did not provide sufficient resources for community support, contributing not only to higher forensic admission rates but also to longer lengths of stay. Due to resource constraints, clinicians are increasingly reporting challenges in liaising with general mental health services, which is making it more difficult to discharge patients into the community. In another qualitative study by Holley et al. (25), forty forensic patients with a range of diagnoses in medium to high secure forensic units in England were interviewed about the factors influencing their length of stay. The majority of respondents identified factors that occurred prior to their admission as influencing their length of stay, including the severity of the offence or their criminal history. Some respondents attributed their length of stay to the structure or organization of the treatment system, which they felt they had no control over. Examples included a change in treating physician and the resulting adjustments to discharge plan requirements, or the lack of a suitable facility to which the patient could be transferred.

### 1.3 Aim of our study

The influence of forensic psychiatric settings on length of stay has been a relatively neglected area of research. The aim of this study was to determine whether there are differences in length of stay between forensic psychiatric units, holding patient characteristics constant. This will help to determine whether the observed differences in treatment are due to patient-related and institutional factors, as opposed to individual factors alone.

#### 1.3.1 Hypothesis

Our study is an exploratory effort to improve and extend the widely accepted prognostic model of length of stay, which focuses primarily on identifying and describing person-related predictors. Our extension includes setting variables that have been relatively under-researched but may significantly influence the length of stay for patients under § 63 of the German Criminal Code. In light of the existing literature, we put forth the following hypothesis (1): it is hypothesized that patient-related factors, including age, severity of offence, substance use disorder and other relevant variables, influence the length of stay in forensic hospitals. Given the limited research on institutional influences, (2) it is further hypothesized that variation in length of stay is significantly influenced by differences between hospitals, independent of patient-related factors. (3) It is also hypothesized that the structural characteristics of hospitals have a significant effect on length of stay.

## 2 Materials and methods

### 2.1 Data

The dataset originates from a forensic documentation system funded by the government of Baden-Württemberg (BW). The system includes socio-demographic characteristics, treatment details, legal issues and psychiatric, forensic and substance abuse history. The data set covers all admissions to the forensic psychiatric system in BW and is compiled annually (reporting date 31 December) by clinical staff (psychologists, doctors, social workers). A glossary is provided to assist the forensic therapists in understanding the terminology used, thereby increasing consistency and reliability. The entries are made by the patient’s primary therapist, who is designated by the clinic for each patient and acts as the patient’s main contact among therapists. The entries are then checked by the primary therapist and a team of specialists. The entries are also electronically checked for plausibility by a team of specialists who regularly ensure valid data collection. The entries are then approved by the departmental medical directors after validation by three professionals from different disciplines (psychologists, data managers, medical officers). Data were collected in accordance with EU regulations (EU 2016/679) and the German Federal Data Protection Act, including specific provisions for mental health data. Data were anonymized before being used for research purposes. The year of discharge is used as the entry for the dataset. Variables for hospitals were extracted from medical controlling occupancy statistics or aggregated from the actual dataset.

### 2.2 Sample

To concentrate on the impact of institutional factors and eliminate the influence of other diagnoses, we limited our data set to patients with SSD, maintaining a high patient count. This approach aligns with the reality that these patients represent the majority of individuals receiving forensic treatment. The dataset comprises patients discharged from six forensic treatment units between 2011 and 2021 (N = 1050). Of the total number of patients, 702 (67%) were diagnosed with SSD (F20-F29). There are various pathways from admission to discharge from a forensic hospital. In this sample, patients were discharged under Section 67d of the German Criminal Code (N=594), which applies when patients are considered low risk or when forensic placement is no longer appropriate. Therefore, the dataset contains N=594 cases for a total of N=6 hospitals.

### 2.3 Variables

#### 2.3.1 Length of stay

The dataset exclusively comprises patients who have been discharged from the facility. The length of stay was calculated by subtracting the day of forensic admission from the day of discharge. In the event that patients received treatment at more than one of the included hospitals during their care pathway, the total length of stay was adjusted to account for the number of treatments. The exclusive use of discharged cases in our dataset may introduce potential biases. In particular, if hospitals are unwilling or unable to discharge patients within the selected time period of 2011 to 2021, this may introduce a bias in the length of stay, whereby hospitals that retain patients for more than 10 years are under-represented. To address this issue, we examined the proportion of long-stay patients relative to the total number of patients treated in each hospital in 2021. The mean proportion of long-stay patients with SSD across all hospitals is relatively low, at 2.8% (standard deviation = 2.23). Furthermore, a bivariate correlation was calculated to examine the relationship between the mean length of stay for each hospital and the mean proportion of long-stay patients, which demonstrated a strong positive correlation (r = 0.93). This indicates that hospitals with a length of stay below the mean tend to have a lower proportion of long-stay patients. Given the small proportion of long-stay patients with SSD and the strong correlation with the mean length of stay, it can be concluded that the potential bias on length of stay is minimal. The term ‘long-stay patient’ is not universally defined. In the UK, however, the threshold for such a designation varies according to the security level of the hospital. For instance, a patient may be considered long-stay if they have been in medium security for over five years or in high security for over ten years (10). The selection of 10 years is consistent with the findings of an earlier study of forensic psychiatric populations in Germany (18).

#### 2.3.2 Independent variables

In selecting the independent variables, we have adopted a pragmatic approach, focusing on indicators that are accessible, valid, reliable and well documented in the existing literature. The majority of studies include socio-demographic characteristics, such as age, gender, education, homelessness and work experience or employment status, as shown in the recent systematic review by Dima et al. (7). This is despite the fact that such variables are only included for control purposes. In addition, numerous criminal history variables have been documented in the existing literature, including age at first conviction, the number of previous convictions, and a history of substance misuse. In addition, variables such as age at first psychiatric admission, number of inpatient treatments prior to forensic admission and psychiatric history are included.

In the existing literature, there are only a few predictors that attempt to account for institutional effects, such as security level. In Germany, there is a lack of systematic differentiation in security standards between hospitals, in contrast to the UK, where a low, medium and high security approach is employed (10). Furthermore, there are no specialist long-term inpatient units in Baden-Württemberg. All forensic psychiatric units are subject to the same legislative framework and thus have the same care mandate, with the exception of one unit which is excluded from the equation. Consequently, the patient population is approximately the same. As a consequence of the expansion of forensic hospitals, driven by a substantial influx of patients, a number of indicators may have undergone significant changes in recent years. The aforementioned changes, which affect forensic hospitals to varying degrees, are likely to affect the length of stay of patients. One potential explanation for this phenomenon is the elevated stress levels experienced by both patients and staff as a consequence of the increased admission pressure and the subsequent densification of wards. Consequently, the increase in hospital size (in percentage) is employed as a proxy for the stress experienced by patients and staff, with a comparison of the number of beds per hospital from 2011 to 2021. Furthermore, it is hypothesized that the size of the hospital may have an impact on patient outcomes. In smaller hospitals, a more familiar environment may foster stronger interpersonal relationships between patients and staff, which may have a positive effect on the length of stay. In order to capture this effect, we selected hospital size (number of beds) as a proxy and calculated the mean number of beds for each year between 2011 and 2021. Furthermore, it is acknowledged that different forensic hospitals may adhere to diverging cultural practices. It is not uncommon for hospitals to implement more restrictive practices or to delay discharges for a variety of reasons or due to differing beliefs. For instance, patients are typically subjected to a probationary period of six months prior to discharge, which can only be extended in exceptional circumstances. However, some hospitals systematically extend this period, opting to maintain a cautious approach by keeping patients under observation as outpatients for longer periods before fully discharging them. To account for these differences, the variable ‘average length of probationary period before discharge’ per hospital (in months) was included as a proxy for differences in cultural practice.

### 2.4 Data analysis

**Figure 1:**
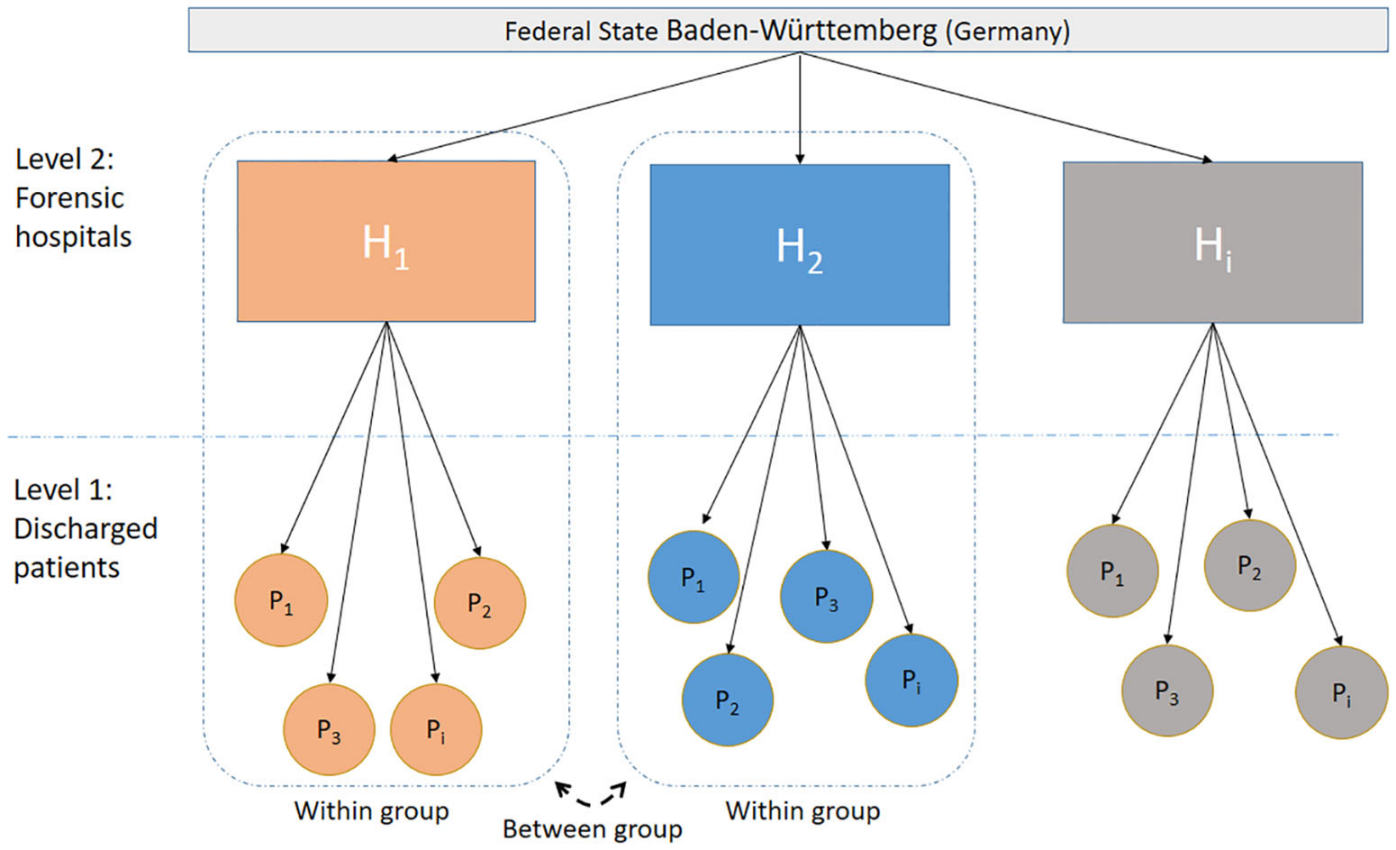
Overview of the hierarchical structure of the linear mixed model used in the analysis, representing the two-level design. Level 1 consists of discharged patients (P₁, P₂, etc.) nested within level 2 representing different forensic hospitals (H₁, H₂, H_i_) within the state of Baden-Württemberg (Germany). The model accounts for both within-group and between-group variability and illustrates the relationship between individual patient outcomes and their corresponding hospital in terms of length of stay.

Multilevel analysis is a specific form of regression analysis and is recommended when there are different levels of abstraction or hierarchy in the data (26). In the case of this study, patients are nested within forensic hospitals, symbolizing a hierarchical structure (see **Figure 1**). In the linear mixed model, the group level is estimated simultaneously with the individual level under mutual control, and the variance explained is split into the proportion of variance within groups (patient level) and the proportion between groups (hospital level). The Literature suggests a minimum of 30 second-level elements and 30 first-level units per element to estimate unbiased parameters (27). However, some authors (28) disagree with the 30/30 rule of thumb and argue that the minimum sample size required depends on the complexity of the model, the number of random effects and the intraclass correlations. In empirical practice, the number of forensic psychiatric units is limited. Hox & McNeish (28) proposed ‘rough guidelines for the minimum number of groups’, in this case the number of hospitals in which patients are nested, and suggested a minimum group size of 20 for cross-sectional samples with fixed-effects multilevel regressions using restricted maximum likelihood (REML); The authors proposed a minimum sample group size of 5, based on simulations conducted by McNeish and Stapleton (29), using the Kenward-Roger correction. It is therefore incumbent on researchers to consider in advance whether to treat predictors as fixed or random effects and which approximation to choose. Random effects are used to estimate variability and differences between different units or subjects within a larger group, as opposed to fixed effects, which capture specific characteristics that remain constant across observations. Theoretically, German forensic hospitals located in the same federal state should not be very different in terms of patient-related predictors. We therefore included each predictor, whether level 1 or level 2, as a fixed effect.

Multilevel analysis typically begins with a null model, excluding the influence of predictors. The analysis provides insight into the distribution of the dependent variable’s variance between the two levels of analysis, patients and forensic hospitals. Furthermore, it indicates the extent to which the contribution to the explanation of the variance is altered by the additional inclusion of the predictors in subsequent models. Intra- and inter-group variance estimates were related to each other using intraclass correlation coefficients (ICC). This is a measure of the proportion of between-group variance, where the proportion of between-group variance is divided by the sum of within-group and between-group variance. The higher the proportion of between-group variance, the more similar patients are in forensic hospitals and the more patients differ between hospitals. Some authors believe that there is no compelling reason to conduct a multilevel analysis for an ICC value below 0.05, as the low proportion of variance at the next higher level does not justify the additional effort of a multilevel analysis (31). Typical values are in the range of 0.10 to 0.25 (32).

## 3 Results

### 3.1 Descriptive results

**Figure 2:**
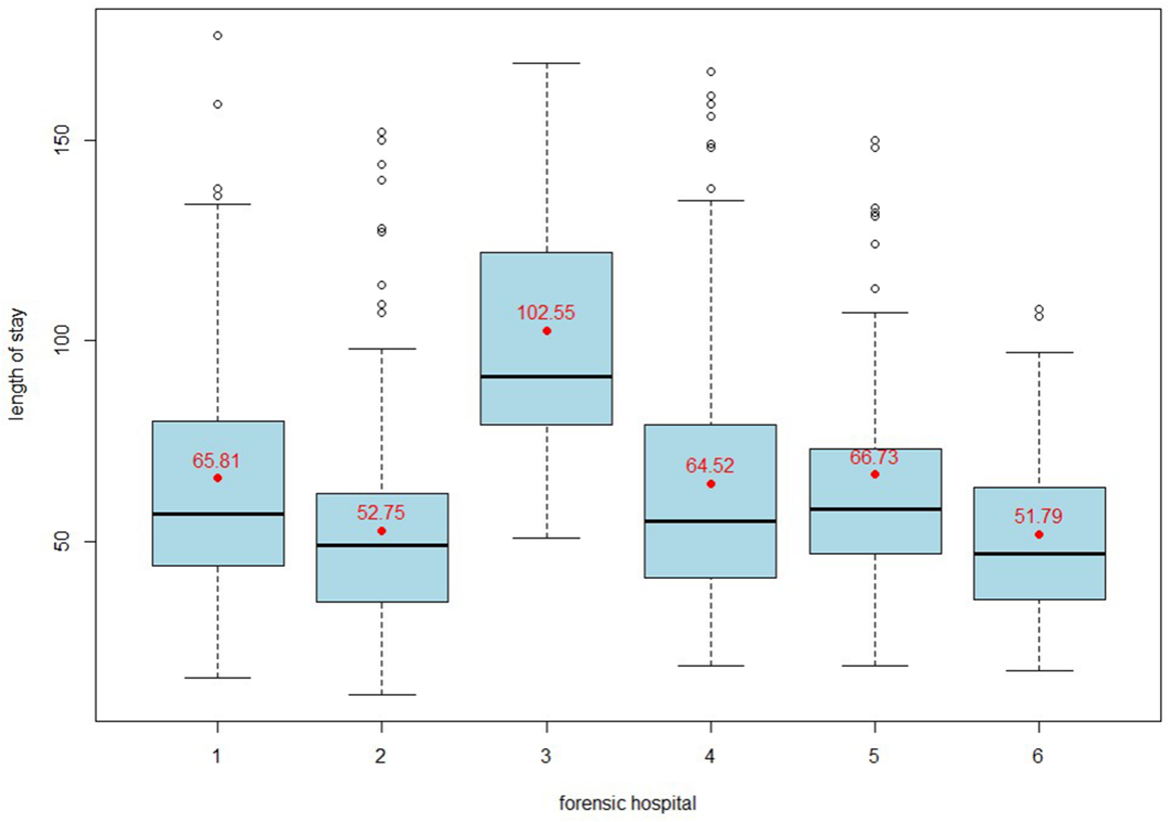
Box plot showing the distribution of length of stay in months in six forensic hospitals. The median length of stay, interquartile ranges and outliers are shown for each hospital, highlighting the variability in length of stay for patients with SSD.

In preparation for linear mixed models, the selected variables were examined in detail according to statistical standards: metric variables were tested for normal distribution and then z-standardized. The only categorical variable, index offence, is dichotomized; the reference category in the multilevel analysis is the index offence of assault, as it is the most prevalent, representing 41.8% of cases. Additionally, a residual category called ‘other offences’ has been created due to the low representation of these types of offences. This category includes offences against the narcotics law, traffic offences and resisting law enforcement officers. The dependent variable for the regression model, length of stay, is ratio scaled and approximately normally distributed. When considering standardization or square root transformation of the Y variable, we deliberately chose not to do so in order to facilitate the interpretation of the effects. **Tables 1**, **2** provide an overview of all the variables included in a linear mixed model. Length of stay, i.e. the period from admission to discharge from the forensic hospital, is expressed in months. **Figure 2** shows the distribution of length of stay by hospital. There are outliers in every hospital (with the exception of hospital no. 3).

**Table 1:** Descriptive statistics of patient-related variables (N = 594).

Binary scale		N	in %
Gender (female)		61	10.9
No German citizenship		157	28.0
Work experience of five years or more		263	47.0
No graduation from school		90	16.1
Homelessness at the time of index offence		43	7.7
Index offence:	Homicide	39	7.1
	Attempted homicide	96	16.2
	Sexual offence	25	4.3
	Assault	248	41.8
	Robbery, threat or coercion	70	11.8
	Arson	60	10.1
	Property crime	25	4.2
	Other crimes	23	3.9
History of substance misuse (comorbid)		333	59.5

Metric scale		Mean	SD
Length of stay (in months)		62.24	30.98
Age at forensic admission (in years)		37.03	11.39
Age at first recorded offence (in years)		30.03	12.78
Number of criminal records		4.43	6.41
Age at first psychiatric admission (in years)		28.18	11.36
Number of inpatient treatments before forensic admission		4.28	3.68

In addition to patient-related characteristics, we also collected hospital-related items from six different forensic units (**Table 2**). Firstly, the size of the hospital in terms of the average number of beds over the period 2011-2021, and secondly, the increase in hospital size per number of beds over the same period. We also aggregated the length of the probationary period before discharge of all patients at hospital level.

**Table 2:** Descriptive statistics of hospital-related variables (N = 6).

Hospital variables (N = 6):	M	SD	Min.	Max.
Hospital size (number of beds)	151.99	69.00	50.00	244.00
Increase in hospital size (in per cent)	31.70	16.56	16.00	81.00
Average length of outpatient extramural testing before discharge (in months)	8.20	2.30	5.80	13.00

### 3.2 Multilevel analysis

A linear mixed model was applied using the ‘lme4’ and the ‘pbkrtest’ packages in R Studio version 4.3.3 (2024–02–29). P-values can be readily obtained through the use of Kenward-Roger approximation, which yields satisfactory acceptable type 1 error rates even for modest sample sizes (30).

#### 3.2.1 Null model

In our study, the null model yielded an ICC of 0.25. This indicates that differences between hospitals account for 25% of the variance in length of stay. This is a remarkably high value that justifies the inclusion of the hospital level in the regression model. The quality of the extended regression model was compared with the null model and the statistical improvement of the model was determined using the model fit evaluation criteria for model adjustment (see **Table 3**). The Akaike Information Criterion (AIC) is a measure of the relative quality of a statistical model for a given set of data. The AIC is derived from the maximum log-likelihood and penalizes models for their complexity. Lower values indicate a better fit. The Bayesian Information Criterion (BIC) is another model selection criterion that is similar to the AIC. The Level 1 and Level 2 models both have lower AIC and BIC values and a higher log likelihood compared to M0, indicating a better fit to the data. Furthermore, the chi-squared test indicates that there is a statistically significant difference in model fit between M0 and M1. However, with a p-value of 0.83, the null hypothesis was not rejected, so there was no statistical evidence that Model 2 provided a better fit to the data than Model 1.

**Table 3:** Model fit evaluation criteria for linear mixed models.

Models	Number of estimated parameters	df	AIC	BIC	Maximum log-likelihood	P-value
M0	3		5730.0	5743.1	-2862.9	
M1	16	13	5654.4	5724.6	-2811.2	≤ 0.01
M2	19	3	5657.9	5741.3	-2810.0	0.83

#### 3.2.2 Level 1 model

Next, individual characteristics were entered into the regression model (level 1 model, as shown in **Table 4**, first column). The model includes variables related to socio-demographics, criminal history and previous psychiatric treatment, which are considered as fixed effects. The increase in the ICC after the addition of patient-level fixed effects indicates that individual differences between patients contributed more to the variability between hospitals than in the null model. However, the increase of 0.03 indicates that there is little difference between forensic hospitals in terms of the patient-related predictors included in the model. This supports Hypothesis 2, that variation in length of stay is significantly influenced by variation between hospitals, independent of patient-related variables. The coefficient of determination (pseudo) R² of the total effects indicates that 36% of the variance in length of stay can be explained by Model 1. At the same time, 12% of the variance can be attributed to individual patient characteristics.

**Table 4:** Linear mixed models (null model; level 1 model; level 2 model).

		*Null model*		*Level 1 model*		*Level 2 model*	
		Coef.	P	Coef.	P	Coef.	P
Estimate		66.88	**	62.34	**	59.71	**
Individual level variables (level 1): N = 594							
Sociodemographic:							
Age at forensic admission (in years)				-6.30	**	-6.30	**
Gender (female)				-0.55	n.s.	-0.52	n.s.
No German citizenship				-0.82	n.s.	-0.87	n.s.
Work experience of five years or more				-2.11	n.s.	-2.14	n.s.
No graduation from school				7.74	**	7.66	**
Homelessness at the time of index offence				6.81	n.s.	6.85	n.s.
History of crime							
Index offence: (ref. category assault)	Homicide			35.10	**	35.04	**
	Attempted homicide			4.02	n.s.	3.95	n.s.
	Sexual offence			13.57	*	13.30	*
	Robbery, threat or coercion			-5.68	n.s.	-5.71	n.s.
	Arson			8.58	*	8.49	*
	Property crime			-4.35	n.s.	-4.23	n.s.
	Other crimes,			-0.87	n.s.	-1.05	n.s.
Age at first recorded offence (in years)				-0.56	n.s.	-0.58	n.s.
Number of criminal records				-0.84	n.s.	-0.90	n.s.
Psychiatric pre-treatment							
Age at first psychiatric admission (in years)				1.53	n.s.	1.54	n.s.
Number of inpatient treatments				1.83	n.s.	1.83	n.s.
History of substance misuse (comorbid)				1.08	n.s.	1.05	n.s.
Variables at hospital level (level 2): N = 6							
Hospital size (number of beds)						-11.95	n.s.
Increase in hospital size (in percent)						-8.54	n.s.
Average length of probationary period before discharge (in months)						3.40	n.s.
Parameter							
Fixed effects (Pseudo-R²)				0.12		0.14	
Total effects (Pseudo-R²)		0.25		0.36		0.49	
Intraclass correlation coefficient (ICC)		0.25		0.28		0.41	

Significance: ** p ≤ 0.01; * p ≤ 0.05; n.s. p≥0.05.

Looking at the individual predictors and their respective coefficients, it becomes apparent that the effect sizes vary considerably. However, only a few of the effects in the model are statistically significant. The results indicate that age at admission, lack of education and the seriousness of the offence have a significant effect on the length of stay. This is particularly evident for homicide, sexual offences and arson. Homicide has the greatest impact on length of stay; the estimated increase in length of stay due to homicide is 35 months. The length of stay is increased by 13 months for those who have committed a sexual offence. Older age at admission is associated with shorter length of stay. In addition, not having completed education is also associated with a longer length of stay. These results support the hypothesis that certain patient-related characteristics have a significant impact on length of stay (hypothesis 1).

#### 3.2.3 Level 2 model

In a further step, we incorporated the predictors at the hospital level to obtain model 2. The effect sizes and p-values of the patient-level predictors remained largely unchanged. At the hospital level, hospital size has the greatest impact. The model indicates that a larger number of beds is associated with a shorter length of stay. Hospitals that have expanded their bed capacity in the past decade tended to discharge patients earlier. The length of the probationary period before discharge has the least impact. However, all three hospital level predictors have a p-value above 0.05, indicating that they are not statistically significant. The high p-values are likely due to the small number of hospitals included in the study. The R² for the fixed effects in model 2 is only slightly higher than the R² from model 1. However, the ICC has increased from 28% (model 1) to 41% (model 2). This observed increase suggests that the hospital level predictors did not significantly alter the variance explained by the fixed effects. Hypothesis 3, which proposed that the structural characteristics of hospitals have a significant effect on length of stay, must therefore be rejected.

## 4 Discussion

It is important to note that the study was not designed to compare or evaluate the effectiveness of regional forensic psychiatric services. Nevertheless, the findings should encourage a greater focus on the forensic psychiatric environment, the differences between hospitals in length of stay and the effects of the institution. However, we employed a multilevel analysis to control for patient predictors while examining the influence of the hospital in order to gain a comprehensive understanding. The model accounts for 49% of the total variance in the length of stay of patients admitted to forensic hospitals, as indicated by the coefficient of determination (R²). Our hypothesis was that patient-related factors predict the length of stay. The results of our regression analysis are consistent with the findings of the majority of previous studies. The severity of the index offence, particularly homicide, sexual offences and arson, has a significant effect on length of stay (20). This effect is present in all hospitals and contributes to longer stays. There is currently no empirical evidence of a relationship between the severity of the index offence and the severity of mental illness. Consequently, lack of responsibility for the offence implies impunity and should not affect length of stay. Given the lack of empirical proof, it seems reasonable to assume that the purported patient-related factor of the severity of the index offence has a cross-hospital effect that could, at least in theory, be attributed to external circumstances rather than success in treatment. Furthermore, the correlation between lack of education and length of stay is a notable finding, suggesting that a significant proportion of patients requiring long-term forensic psychiatric care have significant educational needs. Addressing these needs may alleviate many of the difficulties these patients face on eventual discharge, and prolonged hospitalization offers the potential to improve educational attainment. There is a high prevalence of comorbidity between substance use disorders and SSDs (33). In BW, 59% of patients with a SSD discharged from hospital had a history of substance use (34). However, there is no empirical evidence of an association between a history of substance abuse and length of stay in this study. The available evidence does not support the hypothesis that other socio-demographic factors are associated with length of stay. This finding is consistent with the results of a recent systematic review (7). The only exception to this is age at admission, which has a relatively small effect size. In contrast to the findings of the Czech study (9), which indicated that older age was associated with longer length of stay, the results of the present study suggest that older age at admission is associated with shorter length of stay.

The findings indicate that the institution responsible for forensic treatment has a notable influence on the length of stay. The high ICC of 41% in the level 2 model serves to illustrate the extent to which the length of stay of individual patients is influenced by the hospital in which they are treated. This finding supports the second hypothesis, namely that the observed variation in length of stay is significantly influenced by differences between hospitals, independent of patient-related factors. A null model was constructed to eliminate the potential influence of temporal or period related factors. The intraclass correlation coefficient is 1%, indicating minimal variation in length of stay within the data period.

The third hypothesis is challenging to confirm and requires a more nuanced analysis. Although there are evident discrepancies between hospitals that cannot be attributed to patient-specific factors or outliers, indicating potential variations in the manner in which discharge is managed in forensic hospitals, the predictors at the hospital level remain uncertain. The limited number of observations at the hospital level has an impact on the statistical significance of these predictors and the overall quality of the model. Consequently, it is not possible to discount the potential impact of factors such as hospital size, growth in hospital size and probationary period length prior to discharge. Nevertheless, the evidence provided by our linear mixed model is insufficient to confirm these predictive effects. It is recommended that these predictors be included in future studies, as they are relatively straightforward to collect as structural variables.

The legal framework for forensic psychiatry in Germany is firmly rooted in criminal law. Although clinical expertise plays a crucial role, the decision to release a patient is ultimately taken by the court. The influence of the judicial system cannot be excluded and must be taken into account in any analysis. Each court has its own regional jurisdiction and several courts may cover the catchment area of a single hospital. To examine the influence of the court, a *post-hoc* analysis was performed using a null model in linear mixed models for different courts (N = 17).

The intraclass correlation coefficient is approximately 12%, which indicates a notable impact of the judicial system on the length of stay. Furthermore, several legal factors contribute to this effect. These include delayed approval of the first external relaxation of exit privileges by the prosecution offices involved, less favorable forecasts by external (independent) experts who are reluctant to take any residual risk, and cautious decisions by the courts in the case of capital offences. This is because the recidivism of released forensic patients can provoke a public reaction that is challenging to counter with rational arguments, whether from a legal or a forensic-therapeutic perspective.

## 5 Limitations and outlook

Although the study is distinctive in its approach to accounting for hospital variation and including hospital level effects on length of stay for forensic psychiatric patients, it is important to acknowledge the presence of certain limitations. In particular, certain patient characteristics, such as GAF scores (35) and the chronicity of SSD, which may influence outcomes, were not included in the analysis. Furthermore, the study was constrained by the limited access it had to relevant structural data from the hospitals. It is therefore recommended that this research be regarded as a preliminary step towards the identification of the institutional characteristics that explain variations in length of stay. Further research should consider the impact of both structural and cultural aspects of hospitals, including architectural design (36, 37), on patient outcomes. The impact of cultural practices within institutions is difficult to quantify, indicating a need for theoretical frameworks from organizational theory to inform future empirical studies. In the qualitative study by Holley et al. (25), forensic patients attributed their length of stay to the structure and organization of the treatment system, which they felt they had no control over. The respondents identified the turnover of doctors and nurses as a significant factor necessitating the revision of discharge plans. In order to gain a deeper insight into this phenomenon, it would be advantageous to operationalize the concept of treatment team turnover and incorporate it into future regression models. Furthermore, institutional disparities may be attributable to external (regional) constraints, such as the availability of aftercare facilities. A national survey of forensic hospital directors (38) revealed that a lack of aftercare can complicate and delay discharge. Furthermore, forensic units may have varying access to residential facilities. The availability of a secure place in a mental health residential facility is associated with an increased likelihood of successful discharge. In practice, some mental health residential facilities may exclude patients with comorbid substance use disorders from the outset, thereby making it more difficult to identify an appropriate mental health residential facility and resulting in avoidable ‘delayed discharge effects’ (39). The intertwined nature of criminal law and forensic psychiatry further complicates the separation of hospital and court influences, underscoring the need for comprehensive research that encompasses both domains. A further potential limitation is that patients may have been treated in more than one forensic hospital as part of their care pathway. The proportion of patients for whom this applies is relatively low (7%), and it is therefore unlikely to have a significant impact on the average length of stay within any single hospital. In principle, limiting the analysis to one federal state (BW) can be seen as an advantage, as it avoids having to take into account the numerous other influences, in particular differences in the federal legal framework. However, there are only six forensic psychiatric hospitals in the state, which affects the statistical power of the study. Therefore, larger multicenter studies are required to gain a deeper understanding of the impact of forensic hospitals on length of stay.

## 6 Conclusion

The existing literature has mainly focused on patient-related characteristics as predictors of length of stay in forensic psychiatric settings. Consistent with some findings, our study identifies a significant factor with a strong effect across hospitals: longer stays are observed for patients involved in severe crimes such as homicide and sexual offences. However, there is a notable lack of attention in the literature to the specific institutional characteristics of forensic psychiatric settings and their effect on length of stay. By focusing on patients with schizophrenia spectrum disorders (SSD), this study isolates institutional effects from potential confounders, allowing for a more rigorous analysis. Our results show striking variation in length of stay between hospitals, even after controlling for individual patient variables. This suggests that there may be previously unrecognized institutional factors influencing length of stay over and above patient characteristics. Although the study does not clearly identify specific institutional predictors, possibly due to the small sample size (N = 6), it highlights the role of hospital-specific characteristics in influencing length of stay. We suggest that the hospital structure and ‘discharge culture’ of a forensic hospital, as well as external conditions (e.g. aftercare and court decision) may act as barriers to discharge. This work should be seen as a step towards broadening the current “patient-centered perspective”. It serves as a prelude to further exploratory studies aimed at identifying the institutional characteristics that contribute to differences in length of stay.

## Data Availability

The original contributions presented in the study are included in the article/supplementary material. Further inquiries can be directed to the corresponding author.
